# Titanium for Orthopedic Applications: An Overview of Surface Modification to Improve Biocompatibility and Prevent Bacterial Biofilm Formation

**DOI:** 10.1016/j.isci.2020.101745

**Published:** 2020-10-28

**Authors:** James Quinn, Ryan McFadden, Chi-Wai Chan, Louise Carson

**Affiliations:** 1 School of Pharmacy, Queen's University Belfast, Medical Biology Centre, 97 Lisburn Road, Belfast BT9 7BL, UK; 2 School of Mechanical and Aerospace Engineering, Queen's University Belfast, Ashby Building, Stranmillis Road, Belfast BT9 5AH, UK

**Keywords:** Orthopedics, Microbiofilms, Biomaterials, Surface Science

## Abstract

Titanium and its alloys have emerged as excellent candidates for use as orthopedic biomaterials. Nevertheless, there are often complications arising after implantation of orthopedic devices, most notably prosthetic joint infection and aseptic loosening. To ensure that implanted devices remain functional *in situ*, innovation in surface modification has attracted much attention in the effort to develop orthopedic materials with optimal characteristics at the biomaterial-tissue interface. This review will draw together metallurgy, surface engineering, biofilm microbiology, and biomaterial science. It will serve to appreciate why titanium and its alloys are frequently used orthopedic biomaterials and address some of the challenges facing these biomaterials currently, including the significant problem of device-associated infection. Finally, the authors shall consolidate and evaluate surface modification techniques employed to overcome some of these issues by offering a unique perspective as to the direction in which research is headed from a broad, interdisciplinary point of view.

## Introduction

As human life expectancy continues to rise, so too does the geriatric population, who are at an increased risk of developing chronic musculoskeletal conditions such as osteoarthritis. It is estimated that as much as 15% of the population suffers from osteoarthritis (Johnson and Hunter, 2014). Furthermore, another health concern in developing countries is the rising prevalence in obesity, a modifiable risk factor that has a detrimental impact on musculoskeletal health (Malnick and Knobler, 2006). Mass commercialization and technological advances over the past thirty years have shifted the dynamic of society toward a more sedentary lifestyle, which is associated with increased body mass index (BMI), a risk factor for numerous diseases, including osteoarthritis in weight-bearing joints such as hips and knees (Musumeci et al., 2015).

When treatment options such as physiotherapy and analgesia can no longer manage the condition, arthroplasty (joint replacement) is a surgical intervention utilized to relieve pain and restore functionality of the joint, in so doing also improving the patient's quality of life. In 2018 the National Joint Registry recorded 92,874 total hip replacements (THRs) and 99,093 total knee replacements (TKRs) throughout England, Wales, Northern Ireland, and the Isle of Man (National Joint Registry, 2019). These figures show no signs of decreasing, with THR and TKR procedures projected to rise by 137% from 2005 to 2050 in the United States (Kurtz et al., 2007). Depending on the complexity of the operation and the surgical technique used, the average total hip arthroplasty can cost upward of $20,000 per procedure (Miller et al., 2019). Total hip arthroplasties (THAs) help patients by not only reducing chronic pain but improving their mobility. Patients who undergo a total hip replacement have a significantly improved EQ-5D score after surgery, a standardized measure of general health (Jansson and Granath, 2011).

The arthroplasty procedure involves the insertion of a “biomaterial” into the affected tissue to replace and mimic the diseased bone. The American National Institute of Health defines a biomaterial as “any substance […] which can be used for any period of time, which augments or replaces partially or totally any tissue, organ or function of the body, in order to maintain or improve the quality of life of the individual” (National Institutes of Health, 1982). Expectations for the lifespan of total hip/knee replacements are now outdated, as the patient's own lifespan may outlast that of the implant. Improvements in human life expectancy have now created the necessity for arthroplasties to last much longer. In fact, younger obese patients in need of an osteoarthritis-related hip replacement may require their prosthesis to last fifty years or more.

**Figure 1:**
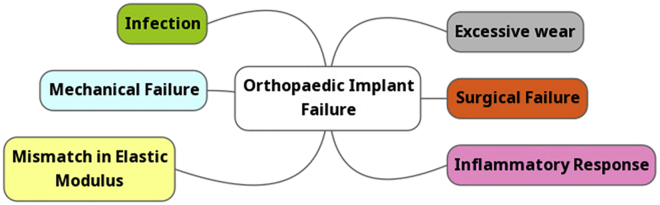
Common Causes of Orthopedic Failure

A recent publication has suggested that only 58% of patients can expect their hip replacement to last for 25 years (Evans et al., 2019). Revision surgery is often required in the occurrence of implant failure, some of the causes of which are shown in Figure 1 and is undesirable for several reasons. Secondary revision surgeries are costly, have a relatively low success rate, and are painful for patients. In addition, patients are up to thirteen times more likely to develop an implant-associated infection, often requiring long-term broad-spectrum antibiotics that could contribute to the urgent threat of antibiotic resistance (Voigt et al., 2016). Currently the National Institute of Clinical Excellence in the UK recommends total hip arthroplasties only in patients with end-stage arthritis, stipulating that the arthroplasty used have 10-year revision rates of 5% or lower (Kandala et al., 2015). In light of this, innovation of biomaterials such as titanium is required if we are to reconsider an even lower revision rate benchmark.

## Titanium as an Orthopedic Biomaterial

### A Brief History of Biomaterials

The use of biomaterials dates back to antiquity, with animal tissue being used by the Egyptians as a suture to seal wounds (Ige et al., 2012). In ancient Phoenicia, artificial teeth were attached to normal teeth using gold wire (Heness and Ben-Nissan, 2004). The titanium was discovered in 1791 by mineralogist William Gregor, a discovery that would later prove to revolutionize the field of orthopedic biomaterials (Lahori et al., 2015). However, the earliest recorded attempts at hip replacement occurred in 1891 and used ivory to replace the femoral heads of patients whose hip joints had been damaged by tuberculosis (Knight et al., 2011). It was not until the mid-twentieth century that the modern interpretation of the metallic hip implant came into fruition, with Sir John Charnley pioneering the modern hip replacement in the 1960s (Wroblewski, 2002). Since then, the global orthopedic implant market has grown substantially and is forecasted to hit USD 8.97 billion by 2025 (Avinash, 2019).

### Titanium Extraction Process

Titanium is the ninth most abundant element within the Earth's crust; however, it is rarely found in its pure form, with the vast majority of titanium being present as insoluble oxides. The most common oxide present in the environment is titanium dioxide (TiO_2_), examples of which include the minerals rutile and anatase (Zierden and Valentine, 2016). Once the mineral ore is acquired, titanium undergoes a series of processes before it is suitable for biomedical application. The mineral ore is reduced to form sponge typically using the Kroll process, which involves the reduction of TiCl_4_ present in the mineral using Mg. The sponge is then subsequently melted to form ingot (Nakamura et al., 2017), which can then be milled and fabricated into various products; the choice of manufacturing process employed varies depending on how the titanium alloy will be utilized. Indeed, the fabrication treatments are used to manipulate the titanium microstructure such that the finalized product possesses desirable metallurgic properties for industrial use, including within the medical devices and biomaterials industry.

### Titanium Grades

Titanium exists in many distinct phases and alloys; therefore, it is not surprising that it can be classified into a variety of different ways. Firstly, titanium and its alloys may be divided into different grades. Around forty to fifty grades are in use, although only a select few are recognized and specified by the American Society for Testing and Materials (ASTM). Commercially pure titanium (cp-Ti) is defined as titanium that consists of <1% of other alloying elements. Cp-Ti constitutes the first four grades of titanium and differ in their impurity content and metallurgic properties. Common impurities include carbon, iron, and oxygen, which are introduced during the manufacturing process.

Cp-Ti is generally lower in strength than the higher, alloyed grades. The most common alloyed grade is grade 5, which is Ti6Al4V. Ti6Al4V has excellent biocompatibility coupled with superior mechanical properties, and it alone makes up more than 50% of all titanium alloys used commercially (Gupta and Laubscher, 2016). A comparative table between the commercially pure Ti grades and Ti6Al4V is shown in Table 1 (Donachie, 1988, Oldani and Dominguez, 2012).

**Table 1:** Properties of Titanium Grades 1–5 from ASTM F 67 and ASTM F 136

	% of Impurity Present					Tensile Strength (MPa)
ASTM Grade	N	C	H	Fe	O	
1	0.03	0.1	0.015	0.2	0.18	240
2	0.03	0.1	0.015	0.3	0.25	345
3	0.05	0.1	0.015	0.3	0.35	450
4	0.05	0.1	0.015	0.5	0.40	550
5	0.05	0.08	0.0125	0.25	0.13	860

### Titanium Phases

**Figure 2:**
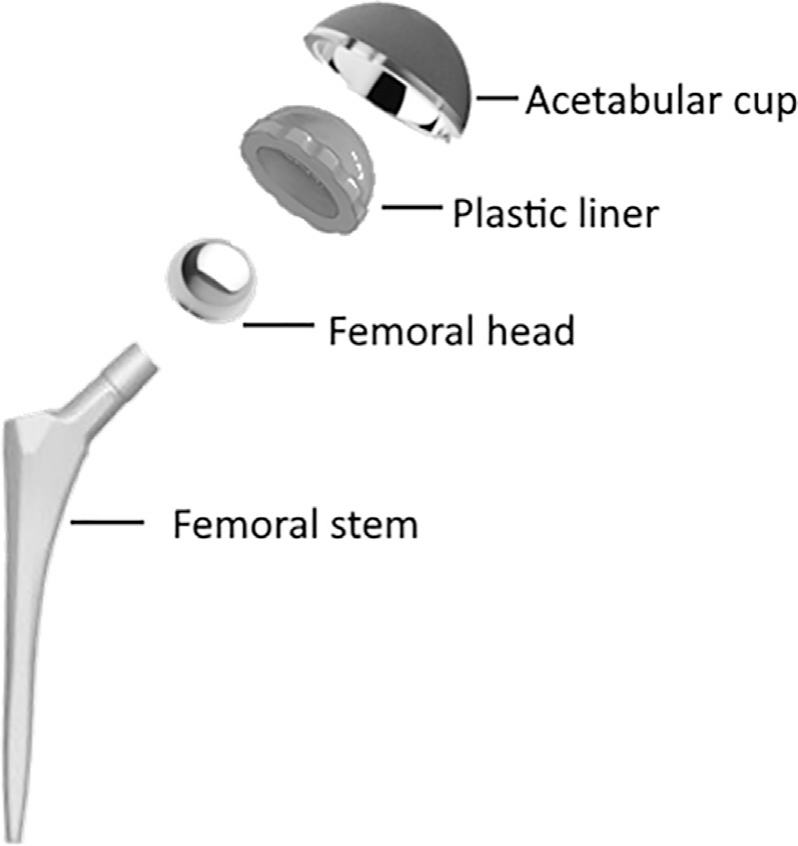
Components of a Total Hip Arthroplasty

Another classification system is based on that fact that pure titanium can be found as two distinct allotropes. At lower temperatures titanium exists in α-phase, which has a closed hexagonal crystal structure; however, above 883°C it exists in β-phase, which has a body centered cubic structure. A schematic of this transformation is shown in Figure 2. The temperature at which titanium moves from the α-phase to the β-phase is known as the β-transus temperature. By modifying the elemental composition of titanium alloys, the β-transus temperature may be altered. α-stabilizers (Al, N, O) increase the β-transus temperature, whereas β-stabilizers (V, Nb, Cr, Fe) reduce the β-transus temperature. Neutral stabilizers (Sn, Zr) have negligible effect on the temperature at which allotropic transformation occurs (Li et al., 2014).

Titanium alloys can be classified into several categories based on their crystalline form. α-alloys contain predominantly α-stabilizers, near-α alloys contain 1%–2% β-stabilizers, α-β alloys contain 10%–30% β-phase upon heating, and metastable-β alloys consist predominantly of the β-phase (Raghunathan et al., 2010). The different categories of titanium all possess their own unique metallurgic properties that can be exploited for use in the biomedical setting. α-alloys possess excellent corrosion properties; however they have relatively low tensile strength compared with α-β and β-alloys (Banerjee and Williams, 2013). α-alloys do however possess higher creep strength than their α-β alloy counterparts (Warlimont and Martienssen, 2018). α-alloys are primarily used in the chemical and engineering industry as their properties are more suited within that sector.

The majority of titanium alloys used within the biomedical field are either α-β or metastable-β alloys, as they tend to satisfy most of the characteristics desired for an implantable medical device. Ti6Al4V and Ti6Al7Nb are regarded as two of the most commonly used titanium α-β alloys in practice (Elias et al., 2008). It is therefore understandable as to why a great deal of attention has recently been focused into the investigation of metastable-β Ti alloys with the intention of discovering novel approaches to improve *in vivo* performance of titanium-based medical devices (Chan et al., 2016; Donaghy et al., 2020; Kopova et al., 2016; Yamako et al., 2017).

### Titanium for Medical Device Applications

The manufacturing of titanium products has advanced substantially following the end of the second world war, and titanium has now been commonplace for use in the biomedical industry for well over half a century (Hussain et al., 2019). Titanium's impressive tribological properties and ability to osseointegrate make it an attractive candidate for a variety of clinical applications including maxillofacial surgery and joint prostheses. One of the most notable uses is the artificial hip, which consists of several components, each made with a different material with corresponding desirable properties that meet the functional needs of that component. Titanium is often used as the femoral stem (as shown in Figure 2) but can also be used in other components.

Alternative clinical uses for titanium include knee replacements, in which titanium is used to replace the autogenous tibial component of the joint. Dental and maxillofacial applications include replacement of missing teeth in edentulous patients using titanium to act as a screw to hold the prosthesis in place or plates and screws to repair maxillofacial deformities and traumatic injuries. Titanium's use as an implanted medical device is incredibly diverse ranging from pacemakers to even cochlear implants (Sculco, 1995, Patel and Gohil, 2012). Currently 70%–80% of all implant materials currently used are metallic, many of which are fabricated from titanium (Mitsuo, 2011).

### Properties of Titanium and the Physiological Environment

**Figure 3:**
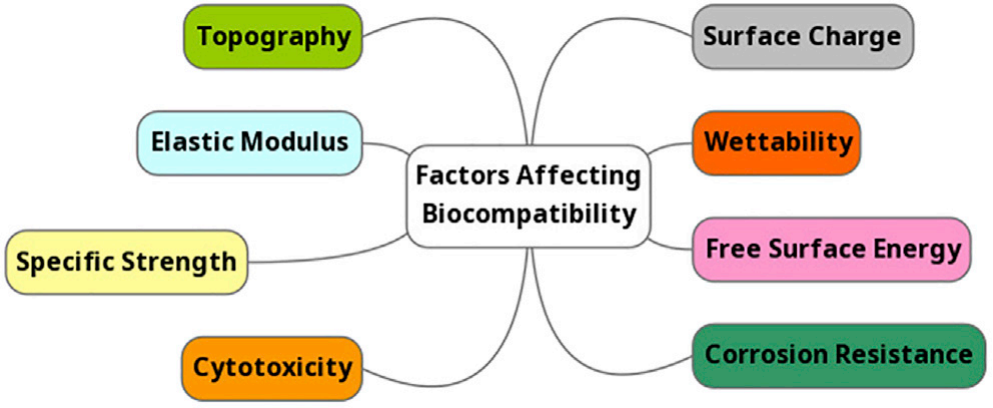
Factors Affecting the Biocompatibility of Titanium

A summary of the factors affecting biocompatibility of titanium is shown in Figure 3, all of which will be discussed in turn.

#### General Biocompatibility

Arguably the most important characteristic of a biomaterial is the need for *in vivo* biocompatibility. There is no universally accepted definition for what biocompatibility means, but “the ability of a material to perform with an appropriate host response in a specific application” is a definition able to encompass “biocompatibility” in the broadest sense of the term (Nair and Laurencin, 2007). The exact parameters required to facilitate osseointegration remain to be found, although a number of important factors have been elucidated (Donaghy et al., 2019). Titanium and its alloys are considered to have excellent biocompatibility among bioimplantable metals. The previously discussed TiO_2_ layer is in part to thank for this, shielding the titanium from the osseous environment with a bioinert oxide layer.

#### Corrosion Resistance

An important characteristic metallic biomaterials must possess is corrosion resistance. Biomaterials will be exposed to the biological milieu of the body, which encompasses interstitial fluid rich in electrolytes (Na^+^, K^+^, Cl^−^, PO_4_^-^) and plasma proteins, all of which can interact with the surface of the biomaterial. All metals undergo electrochemical corrosion, which can weaken the structural integrity of the implant. Fortunately, when titanium is exposed to the *in vivo* environment, a phenomenon known as auto-passivation occurs, an effect that grants titanium its corrosion-resistant properties. The titanium surface spontaneously forms a protective oxide coating that helps to shield the bulk material from the surrounding biological environment. Titanium has the highest corrosion resistance of the commonly used metals (stainless steel alloys, cobalt-chromium alloys) for implantation. (Interestingly, magnesium's low corrosion resistance has been exploited as a means of developing bioresorbable implants (Schmutz et al., 2018).) This oxide layer, while offering corrosion resistance, is quite thin and offers little protection in potential for the release of titanium wear particles into the adjacent tissue (Li et al., 2010). Wear particles pose a serious problem for orthopedic implant success, and several rejected arthroplasties recovered have found wear particles in the surrounding tissue (Grosse et al., 2015; de Pasquale et al., 2018; Hall et al., 2019; Sakamoto et al., 2016). Furthermore, titanium debris has been found to distribute throughout the body, with traces found in the liver, spleen, and lymphatic system (Urban et al., 2000). Efforts of improving the thickness and stability of the passive oxide layer as a means to prevent or hinder wear debris release have been the focus of some interesting research, which will be discussed in the later sections of this review.

#### Surface Charge

Surface charge also has a significant impact on biocompatibility, albeit the theory behind this is not yet fully understood. Surface charge of a biomaterial will drastically influence the initial plasma protein binding, which occurs during implantation, and in turn this protein conditioning film may effectively “buffer” the charge of the material surface and have a significant impact on the adherence of both bacterial and mammalian cells. These surface-bound host factors may also serve as specific receptors for both tissue cells and colonizing bacteria. Titanium, for instance, has a positively charged surface and will initially form non-covalent bonds with negatively charged proteins such as albumin and fibronectin (Jager et al., 2017, Do Nascimento et al., 2017). Fibronectin has been observed to promote bacterial adhesion, whereas albumin and whole serum appear to inhibit bacterial adhesion to biomaterial surfaces (Ribeiro et al., 2012).

In terms of interaction with the biomaterial itself, most bacterial species within an aqueous solution tend to be negatively charged and so would also preferentially adhere to positively charged surfaces (Katsikogianni and Missirlis, 2004). Similar observations have been made for the attachment of mammalian cells, for example, fibroblast cell attachment has been shown *in vitro* to be more favorable on positively charged titanium (Hamdan et al., 2006).

Evidently, surface charge is paramount in how a biomaterial interacts with plasma proteins and mammalian cells but also influences potential biofilm formation. Careful consideration must be applied to surface modification techniques that intend to manipulate the surface charge of a biomaterial. Research into more practical applications for surface charge modification, as well as understanding the underlying mechanism behind how surface charge affects osseointegration will be of great interest in the development of orthopedic biomaterials.

#### Cytotoxicity

**Figure 4:**
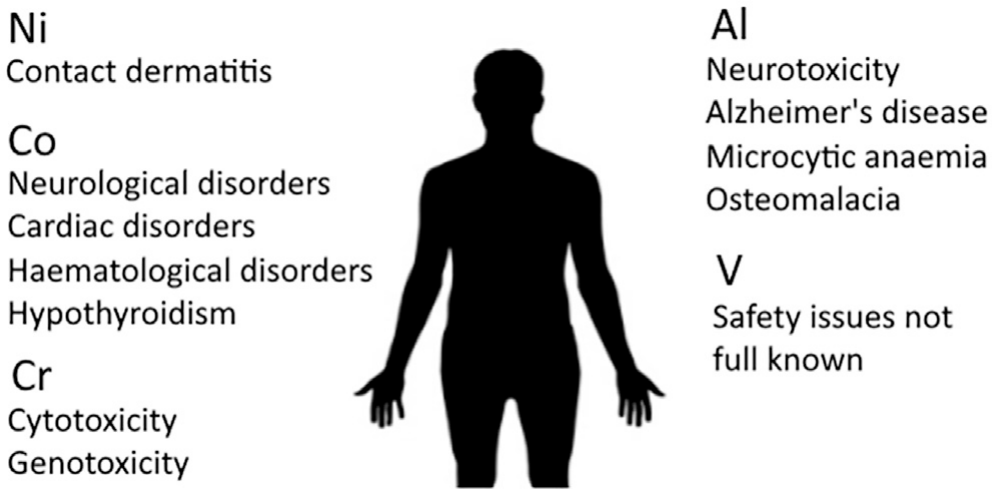
Safety Issues Concerning Elements Commonly Found in Orthopedic Materials

Understandably the chemical composition is of utmost importance in orthopedic biomaterials. Safety issues concerning chemical elements commonly found in orthopedic materials are summarized in Figure 4. The ideal biomaterial should contain no compounds that have cytotoxic or genotoxic potential. In addition, they should not elicit a sustained hypersensitivity reaction within the body. Nickel for instance, is frequently associated with contact dermatitis and is therefore considered incompatible for long-term use as an implant (Chenglong et al., 2007). Commercially pure titanium possesses excellent biological compatibility in relation to other popular metallic biomaterials.

Two of the most commonly used metallic biomaterials are cobalt-chromium alloys and stainless steel (an alloy of carbon and iron with a minimum of 11% chromium content by mass and a maximum of 1.2% carbon by mass) (International Organisation for Standardization, 2014). Although chromium is a naturally occurring micronutrient, it has been shown to have potential genotoxic and cytotoxic effects and may also accumulate intracellularly (Shrivastava et al., 2002). Although cobalt-based alloys are highly resistant to corrosion, and reasonably resistant to fatigue and cracking, wear processes can lead to release of cytotoxic Cr, Co, and Ni ions into the body. This has proven a particular problem with metal-on-metal (MoM) arthroplasties (i.e. hip implants with both ball and socket composed of cobalt-chromium alloy). There are reports of neuropsychiatric morbidities due to cobalt and chromium toxicity following MoM hip implant failure (Green et al., 2017). The use of cobalt also raises issues of biocompatibility that may manifest in a myriad of different ways, including deafness, vertigo, cardiac morbidities, hematological disturbances (polycythaemia), and hypothyroidism (Sansone et al., 2013). For both stainless steel and cobalt-based alloys, wear can produce long-term changes in the metal content of blood and elevations of metallic content in tissues (e.g. kidney and liver) (Eliaz, 2019).

The most commonly used titanium alloy, Ti6Al4V (titanium with 6% aluminum and 4% vanadium), poses its own assortment of problems regarding biocompatibility. A possible association between aluminum and neurotoxicity and/or Alzheimer disease has been suggested in the literature (Kawahara and Kato-Negishi, 2011). Furthermore, aluminum toxicity can also lead to microcytic anemia and osteomalacia (Schifman and Luevano, 2018). Vanadium is a transition metal and is found in trace amounts in the human body. Safety concerns around vanadium exposure have been noted albeit further research into this area is required (Bombač et al., 2007).

Understandably new titanium alloys have been developed in an effort to alleviate biocompatibility concerns, by replacing the potentially hazardous elements discussed prior, with alternative compounds possessing improved safety profiles. Success has been found by incorporating niobium, tantalum, and zirconium into titanium alloys, although unfortunately they are currently much more expensive to synthesize (Liu et al., 2017a, 2017b). For these new titanium alloys to become marketed products, efforts will need to be made logistically to improve the efficiency of production.

#### Topography, Wettability, and Free Surface Energy

The topography of the biomaterial will impact osseointegration; innovative methods have been developed to modify the titanium surface to possess desirable topographical features without affecting the bulk properties of titanium. Li et al. postulated that microtopographical and nanotopographical surface roughness work synergistically to enhance the performance of orthopedic implants (Li et al., 2015). Nanoscale roughness is thought to replicate the intrinsic surface roughness of bone, helping to promote osseointegration (Kane and Ma, 2013). The nanoarchitecture will also influence the adhesion of bacteria, with research again suggesting that nanoscale roughness will hinder bacterial adhesion (Ercan et al., 2011a, 2011b; Truong et al., 2010). What is truly fascinating is that surface topography can be observed in nature as having an antimicrobial/anti-adherent effect. Cicada wings have nanoscale pillar patterns that protect the insect from bacterial onslaught (Diu et al., 2014). Harnessing naturally inspired nanotopography is rapidly gaining momentum as a research avenue for the development of antimicrobial surfaces (Tripathy et al., 2017). A bio-inspired antibacterial nanotopography formed via thermal oxidation on titanium alloy was shown to have modest antimicrobial properties, reducing bacterial viability by 40% (Sjöström et al., 2016), whereas etching techniques used to produce bio-inspired micro- and nano-topographies on aluminum surfaces showed bactericidal activity against 97% of adhered *Escherichia coli*, but disappointingly only 28% of *Staphylococcus aureus* (Hasan et al., 2018). The variability in effect highlights the need to fully appreciated cell-material interactions and to consider that fouling of the bio-inspired surface may negate the antimicrobial efficacy of the surface in question.

The free surface energy of a biomaterial describes the excess energy that exists at the surface in comparison to the bulk of the biomaterial. It can often be used as a predictor of potential interactions between the surrounding environment and the biomaterial. Hydrated titanium surfaces that are able to retain a high free surface energy were found to promote enhanced osteoblast differentiation *in vitro*, and it is suggested that this could in part be the reason for enhanced bone formation that can be observed *in vivo* on titanium surfaces that have had a surface modification technique employed (Zhao et al., 2005).

Wettability is influenced by the free surface energy of the biomaterial and the topography. Wettability describes the interaction of a water droplet with a surface, commonly measured using a tensiometer. A wettable surface is hydrophilic and will result in a contact angle of <90°, whereas a non-wettable surface is hydrophobic and will have a contact angle >90°. Most bacteria are hydrophobic, including *S. aureus,* a commonly implicated pathogen in orthopedic infection (Chan et al., 2017). This means that most bacteria typically prefer to adhere to hydrophobic surfaces and so if surface modification can successfully improve surface hydrophilicity, bacterial adhesion should, in theory, be reduced (Cunha et al., 2016). Superhydrophobic (contact angle >150°), water repellent surfaces are also of interest in the prevention of biofouling. Indeed, in titanium treated with anodization and perfluorooctyl-triethoxysilane, the resulting TiO_2_ nanotubes formed on the surface are and were shown to reduce *S. aureus* adhesion (Tang et al., 2011).

#### Mechanical Properties

The elastic modulus of a material is the resistance of a material to being deformed under stress. One might be led to believe that the ideal orthopedic biomaterial would have a higher elastic modulus (high stiffness) to prevent deformation *in situ*; however, this is not necessarily true. Wolff's law describes how bone will adapt to the load under which it is placed and vice versa. This means that if an orthopedic biomaterial has a much larger elastic modulus than what it is replacing, the surrounding bone will suddenly be placed under much less stress than it typically endures. Consequently, this leads to an increase in osteoclast activity, bone atrophy, and ultimately aseptic loosening (a major cause of implant failure). This phenomenon is commonly referred to in literature as “stress shielding” and is governed by the principles described above (Arabnejad et al., 2017).

**Figure 5:**
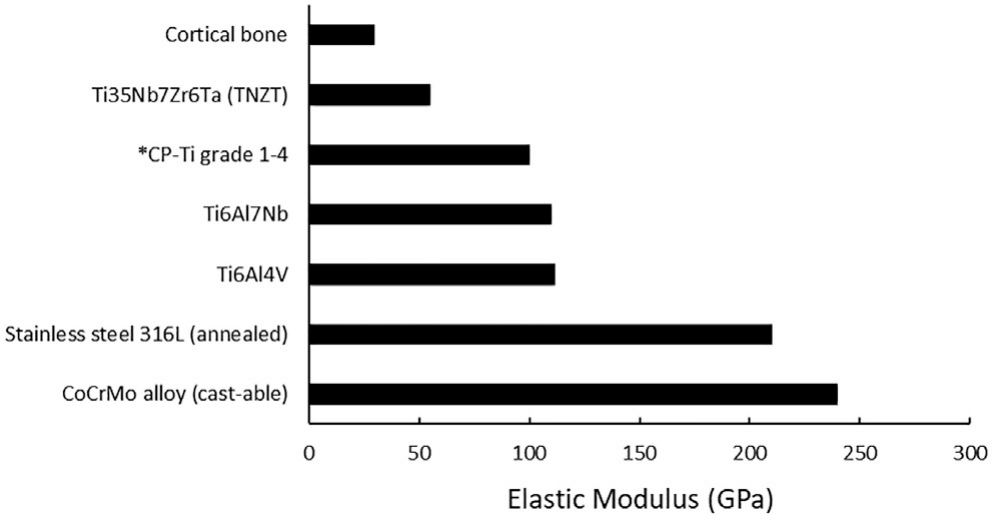
A Comparison between the Elastic Moduli of Various Orthopedic Materials and Cortical Bone ∗Approximate value for CP-Ti

The elastic modulus of bone varies depending on the type of bone (cancellous or cortical) and the direction in which the measurement is taken. Although titanium has a larger elastic modulus than that of the outer cortical bone it replaces in total hip arthroplasties, its elastic modulus is still lower than many of the other metals used in orthopedics as seen in Figure 5 (Bahraminasab and Jahan, 2011; Bahraminasab et al., 2013; Geetha et al., 2009; Niinomi et al., 2012).

Titanium possesses excellent mechanical properties, having the highest inherent specific strength (strength to weight ratio) of any pure metal (Lewallen et al., 2015). This is a highly desirable characteristic especially for an orthopedic biomaterial, which may have to support sustained articulation cycles over a prolonged period of time. Titanium has demonstrated in a clinical setting that it can be successfully used for arthroplasty in obese and super-obese patients (Mclaughlin and Lee, 2014; Castagnini et al., 2019). As mentioned prior in Table 1, the lower grades of Ti have a relatively low tensile strength compared with their alloyed counterparts. Fortunately, the lower Ti grades still surpass the tensile strength of the cortical bone that they are replacing and as such, should be able to sustain repeated articulation cycles as designed.

## Biofilm Formation and Prosthetic Joint Infection

As presented within Figure 1, medical device infection is one of several causes of orthopedic implant failure. Device-associated infection occurs due to the preference of bacteria to exist in sessile, surface-attached communities, known as biofilms, which can establish upon the substratum of the implanted device. When planktonic bacteria (free-moving, single-cellular bacteria) present in the osseous environment attach to the implant surface (therein becoming sessile bacteria) and begin to aggregate, a biofilm is formed. Bacteria that exist within the biofilm are part of a complex, multicellular community enclosed within extracellular polymeric substances (EPS). The EPS produced by bacteria form a “slime layer” around the cells, comprised primarily of water with a variety of polysaccharides, nucleic acids, proteins, and lipids (Flemming et al., 2007). Initial bacteria attachment to a surface consists firstly of a reversible adhesion, followed by a secondary irreversible attachment. Although complete understanding for both of these mechanisms has yet to be unraveled, theories and models have been created in an attempt to explain these phenomena. It is generally accepted that initial reversible attachment consists of the physicochemical, chemical, and physical interactions between a substrate and bacterial cells (Wang et al., 2015; Pulido et al., 2008).

Some of the most common species of bacteria present in biofilms across various medical devices are *Staphylococcus aureus, Staphylococcus epidermidis, Staphylococcus viridians, Klebsiella pneumoniae, Escherichia coli, Escherichia faecalis, Proteus mirabilis,* and *Pseudomonas aeruginosa* (Bjarnsholt, 2013; Chen et al., 2013) The *Staphylococcal* genus is the most common etiological agent responsible for orthopedic infection, with some estimates stating as much as 75.5% of infections involve *Staphylococcal* species (Arciola et al., 2015). Biofilms can be either monomicrobial or polymicrobial, with evidence that even monomicrobial biofilms possess sub-populations with differing phenotypic/genotypic behaviors, despite being the same microorganism. Certain bacterial species may have differing responses to specific antimicrobial agents or immune responses, making them even more challenging to detect and treat (Tande and Patel, 2014; Marculescu and Cantey, 2008). There is also currently no known biomarker that specifically identifies biofilm-mediated infection on dwelling medical devices (Khatoon et al., 2018; Paharik et al., 2016; Tande and Patel, 2014).

Currently, the standard treatment for device-associated infection is the removal of the indwelling implant, debridement of infected tissue, and a prolonged period of antibiotic therapy, before the implantation of a new device (Donlan, 2001; Tande and Patel, 2014; Zimlichman et al., 2013). This has varying difficulty, cost and health consequences depending on the removed device, and severity of the infection. With the threat of both antimicrobial resistance, the emergence of multi-drug resistant pathogens, and the general antimicrobial tolerance observed in biofilm-mediated device-associated infection, it is crucial to understand how an implanted device becomes infected and to address the problem with reduced dependency on antibiotics (Li and Webster, 2017).

### Mechanism of Biofilm Formation

The pathogenesis of orthopedic implant infection is a result of biofilm development upon the surface of the device and can be divided into distinct phases, which will be discussed below. Although a great deal of progress has been made of the past few decades in attempting to understand the etiology of biofilm formation on medical devices, it is important to appreciate the area is still an ongoing area of research. Firstly, upon implant insertion, a “race for the surface” is thought to occur, in which native host cells (fibroblasts, platelets, macrophages) compete with bacteria for initial adherence upon the implanted device (Gristina et al., 1988).

**Figure 6:**
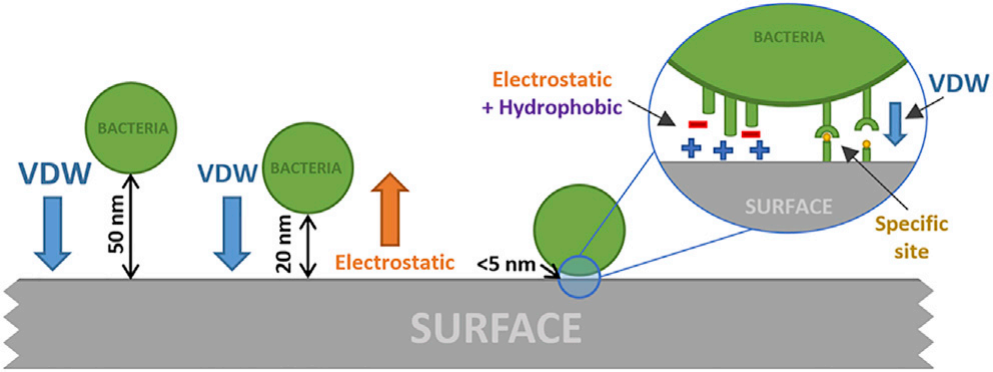
Common Forces of Attraction Experienced between Bacteria and a Solid Surface Namely van der Waals forces (VDW), Electrostatic, Hydrophobic, and Site-Specific Interactions

Bacteria begin to experience non-specific forces of attraction in a significant manner as they come within a few nanometres distance of the surface. At approximately 50 nm from a surface, bacteria will experience van der Waals (VDW) forces of attraction. As the bacteria is about 20 nm from the surface, electrostatic forces of repulsion between the surface and bacteria begin to take effect. Electrostatic forces are dependent on the interaction between the surface, the medium (pH, acid-base interactions etc.), and the bacteria (e.g. at neutral pH, most bacteria are negatively charged) (Hermansson, 1999; Boks et al., 2008). Within 5 nm from the surface, both the VDW and electrostatic forces are at their strongest and a variety of other forces begin to take effect, namely hydrophobic interactions and site-specific interactions. A summary of the forces of attraction experienced by bacteria and implant surfaces is shown in Figure 6.

The hydrophobicity of a surface and/or bacteria is governed by its surface energy, chemistry, and texture, as discussed above in section 2.6.5. Bacteria that are hydrophobic in nature attach preferentially to hydrophobic surfaces and the same is true for hydrophilic bacteria on hydrophilic surfaces (Katsikogianni and Missirlis, 2004; An and Friedman, 1998). Fimbriae, pili, and other surface appendages that exist on the envelope of the bacteria contribute to the hydrophobic effect (Berne et al., 2018). Site-specific interactions occur as physical parts of the bacteria such as the pili or fimbriae begin to attach and form bonds with the material/conditioning film present on the surface. O-side chains of lipopolysaccharides, which can extend from the surface of gram-negative bacteria, can form hydrogen bonds with mineral surfaces (Landini and Zehnder, 2002). As more and more of these physical interactions occur, the bacteria begin to transform from their planktonic state to their sessile state, and colonization of the surface proceeds accordingly.

Following the adhesin-based initial attachment, the pioneering bacteria begin to rapidly proliferate, entering into the log phase of growth. Once environmental factors are appropriate, the bacteria begin to produce EPS, which can facilitate the trapping of nutrients for the bacteria to utilize, as well as improve their overall survivability (Costerton et al., 1999). At this stage the bacteria are sessile and irreversibly attached to the implant surface. As the biofilm continues to develop, subcommunities within the biofilm begin to shift in their phenotype, resulting in the biofilm bacteria adapting a more heterogeneous nature that contributes to the resilient nature of biofilms against antibacterial agents (Dibartola et al., 2017). Cell-to-cell communication within a biofilm is regulated by a phenomenon known as quorum sensing, in which bacteria release particular population density-dependent signaling molecules to coordinate gene expression (Fuqua et al., 1994). Some sessile bacteria present within the biofilm are capable to reverting back to their original planktonic phenotype if environmental conditions change, in a bid to disperse and relocate to colonize a new substratum (Patel, 2005).

### The Difficulties of Biofilm Treatment and Eradication

**Figure 7:**
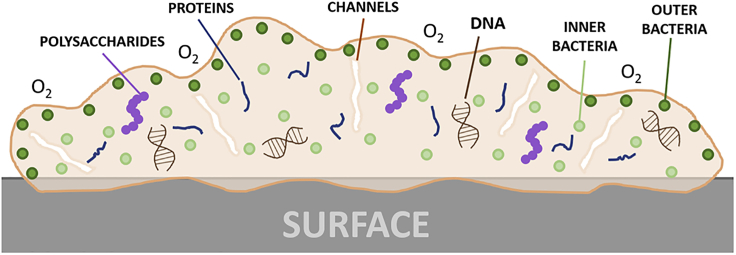
Schematic Demonstrating the Biofilm EPS and the Heterogenic Nature of Bacteria within a Biofilm

The fundamental reason as to why bacteria found within biofilms are so much more resilient to environmental stresses (antibiotics, host leukocytes) is multifactorial, due in part to the surrounding EPS layer, but can also be attributed to phenotypic and metabolic changes in the bacterial cell. Upon attachment upon a biotic or abiotic surface, sessile bacteria begin to promote genes that regulate EPS production at an accelerated rate. As highlighted in Figure 7, this extracellular matrix creates a protective outer “shield” around the bacteria, with channels interspersed throughout the matrix to allow for nutrient transport, whereas simultaneously protecting the bacteria from desiccation (Costerton et al., 1995). In addition, the EPS layer also shields the bacteria from host leukocytes, such as macrophages, rendering them unable to interact with the bacteria, a phenomenon referred to as “frustrated phagocytosis” (Leid et al., 2002). Furthermore, EPS helps to retain extracellular enzymes within close proximity to the bacteria, facilitating the enzymatic degradation and release of essential nutrients (Stoodley et al., 1994).

Once in this state, the sessile bacteria are up to 5,000 times more tolerant to antibacterial therapy than their planktonic counterparts (Bjarnsholt, 2013; Gupta et al., 2015). This is in part due to the EPS layer functioning as a diffusion barrier between molecules (such as antibiotics) and the bacteria within the biofilm, reducing the rate at which the antibiotic can reach the bacteria. As an oxygen concentration gradient exists around the outer parts of the biofilm, wherein the bacteria at the extremities of a biofilm consume the surrounding oxygen, bacteria located toward the center of the biofilm experience a depletion of oxygen. The hypoxic environment results in a much slower metabolic phenotype for these so-called “persister cells,” which are dramatically recalcitrant to the effects of antibacterial agents, which typically exploit the metabolic processes of bacteria (Ghannoum and O’toole, 2004), for example cell wall synthesis, protein synthesis, and DNA synthesis, all of which are essential processes for bacterial growth and proliferation. The persister cell phenotype is considered to be a defense mechanism against adverse environmental onslaught (Leung and Lévesque, 2012). Cell lysis/death can also be mediated within the biofilm itself to produce DNA scaffolds that help the development of the biofilm architecture (Rice et al., 2007; Webb et al., 2003; Desai et al., 2019).

As evidenced throughout this section, biofilm formation is highly complex, with individual bacterial species likely to have their own unique mechanism. For instance, *Staphylococcus* species possess an antigen known as polysaccharide intercellular adhesion (PIA), which is thought to enable intercellular biofilm attachment and mediate biofilm formation (Veerachamy et al., 2014). Ultimately, a great deal more research is required to fully appreciate the underlying mechanisms involved, such that researchers can identify the basis on which novel anti-biofilm strategies can be built.

## Complications Associated with Orthopedic Implants

As with all invasive procedures, arthroplasty is not without risk. Both infectious and non-infectious complications may result in orthopedic implant failure, some of the most common causes of which will be discussed herein. An ideal orthopedic biomaterial would possess both surface and bulk properties to circumvent both of these post-surgical sequelae.

### Infectious Complications

Infection of medical devices constitutes a global health concern as they are highly problematic to diagnose, treat, and are expensive, both in terms of economic cost and quality of life, as they often necessitate further implant revision surgery. Periprosthetic joint infection (PJI) refers to infection of tissue adjacent to the implanted medical device and is a serious complication of arthroplasty. PJIs are not only associated with high morbidity and mortality, they often require complex treatment strategies that often conclude with surgical removal of the affected implant. Infection of the prosthetic joint is the result of bacterial biofilm formation on the device surface.

Diagnosis of infection can often prove difficult, particularly in cases involving low-grade infection, with only joint aspiration able to confidently ascertain if there is evidence of bacterial infection in the joint (Otto-Lambertz et al., 2017). Often PJI diagnoses rely on clinical manifestations experienced by the patient (pain, oedema, impaired joint mobility) and non-specific markers of infection such as raised leukocyte count and raised C-reactive protein levels (Izakovicova et al., 2019). New diagnostic molecular methods that are able to rapidly diagnose PJIs are required. D-lactate is one such biomarker being investigated as a potential diagnostic indicator for PJI (Yermak et al., 2019).

It is thought that during the surgical placement, the implant may be contaminated by transfer of microorganisms from the patient's own commensal skin flora, hence explaining why *Staphylococcus* species represent the pre-eminent source of nosocomial infection. This initial contamination progresses to colonization of the implant surface and biofilm formation (Havard and Miles, 2015), which can subsequently have an adverse effect the functionality of the prosthesis through the perturbation of osseointegration (Cunha et al., 2016). The protective biofilm EPS, combined with metabolic changes in biofilm cells and the unique pharmacokinetic environment of bone tissue (resulting in poor penetration of many antimicrobial drugs), means that these infections are very difficult to treat with conventional antibiotic therapy (Thabit et al., 2019; Ribeiro et al., 2012).

Dissemination of biofilm infection from the surface of the implant to the peri-implant bone and tissues is a concern that must be taken into account in order to optimize the outcomes of surgical debridement and the treatment of PJI. In a study of a porcine model of PJI, bacterial biofilm on the surface of the implant had spread to the surrounding bone within six days (Jensen et al., 2017). There is also evidence to suggest that *S. aureus* may enter and reside within bone canaliculi, effectively establishing a reservoir of bacteria deep within bone tissues and potentially giving rise to chronic or recurrent osteomyelitis (de Mesy Bentley et al., 2017; de Mesy Bentley et al., 2018).

Bone cements (acrylate-based polymers used for fixation of the implant to bone) have been developed to incorporate an antibiotic, typically broad spectrum antibiotics, typically gentamicin, in an attempt to combat PJI (Chang et al., 2013). In recent years cement-less implants have become more popular (due to favourable functional longevity in younger patients). This leads to the necessity for alternative, material- and surface-based strategies to overcome PJI (Giebaly et al., 2016).

Photodynamic therapy as a non-invasive method to treat biofilm formation presents an interesting solution. Mesoporous polydopamine nanoparticles integrated upon titanium surfaces with an attached indocyanine green photosensitizer is able to reduce *S. aureus* biofilm formation by 95% in murine specimens upon exposure to near-infrared, all while supporting osteogenesis and osseointegration (Yuan et al., 2019).

Another promising area of research is the use of bioactive glass (bioglass); the bioglass BGF18 is highly adaptable and can be used to coat titanium surfaces, in doing so, imparting bactericidal properties. Titanium coated with BGF18 displayed significant anti-biofilm activity even at low concentrations (1.34mg/mL) compared with untreated titanium in the initial periods of biofilm formation against both *P. aeruginosa* and *S. epidermidis* (Marques et al., 2020).

Although various research has looked at impregnation of implant surfaces with antibiotics, efficacy and contribution to the ever-growing antibiotic resistance saga is a major concern (Zoubos et al., 2012). Hence, novel approaches involving surface modification of medical devices with the intention of making the surface anti-adherent and/or antimicrobial, while maintaining biocompatibility within the human body, is paramount to improving the health of patients requiring surgical intervention.

### Non-infectious Complications

Aseptic loosening refers to extensive localized bone resorption that results in loosening of the implant and loss of integration. Importantly, by definition, this occurs in the absence of infection. Osteolysis (or active bone resorption) may take the form of focal islands of bone loss without symptoms or extensive and equally distributed around the implant (Sundfeldt et al., 2006). Symptomatic patients may suffer from pain and instability exacerbated by activity and can be just as severe as the osteoarthritic condition that the prosthesis was intended to cure.

There are several theories regarding the causes of aseptic loosening; however, “particle disease”, a term coined by Dr William Harris, is a popular explanation (Harris, 1994; Sukur et al., 2016). Particles generated by wear and/or corrosion of the prosthesis can induce a complex host immunologic response, culminating in osteoresorptive processes predominating over osteogenesis. This can, over time, lead to macroscopic bone defects. The degree of bone loss is in part due to the number and size of the particles. Those in the sub-micrometre range can significantly affect the tissue response, causing initial inflammation and increasing numbers of macrophages and fibroblasts in the joint fluid (Pastides et al., 2013). Intraarticular pressure can also increase and particles can migrate from the area, penetrating between the bone and prosthesis (Mert et al., 2013; Berend et al., 2008; Wong et al., 2011). This causes a membrane of granulation tissue to develop, abundant in fibroblasts, macrophages, and inflammatory mediators. As wear particles continue to be released, inflammation worsens and the neighboring bone is ultimately destroyed (Pap et al., 2001).

Other processes that may cause, or contribute to, aseptic loosening include micromotion and stress-shielding. Micromotion refers to small movements between implant and bone during normal movement within the patient and is undetectable by radiography. This can be detrimental to the healing process and osseointegration, analogous to the situation required for fracture repair, where stability is essential for healing (Sundfeldt et al., 2006). Stress shielding is a result of new loading conditions imposed on the bone by the implant. This can lead to bone loss around the implant in areas that are not subject to loading and is a result of bone remodeling, rather than osteolysis (Niinomi and Nakai, 2011).

## Surface Modification of Titanium Orthopedic Materials

A plethora of surface modification techniques have been investigated with the intention of promoting osseointegration and/or exerting an antibacterial effect on titanium implants (Table 2). For the purposes of this review, methods shall be classified as coating and non-coating methods. It is often quite difficult to categorize some of these novel techniques as most are still in the preliminary development stage or some fall into both categories. Depending on how they are applied, a method could be employed to coat titanium with a biocompatible layer, and equally, the same method may be used to conduct a non-coated modification.

**Table 2:** A Summary of the Advantages and Disadvantages of Commonly Employed Surface Modifications

Method Classification	Method	Advantages	Disadvantages	References
Coating	TiN coatings	Increased wear resistance Antibacterial properties	Clinical benefit unknown	(Chouirfa et al., 2019; Scarano et al., 2003; Van Hove et al., 2015b; Zhao et al., 2009)
	Plasma spraying	Low cost Long lifespan Improves bone growth Evidence-based success from marketed products	High temperature may affect coating and/or bulk structure	(Shahali et al., 2017; Sun, 2018; Fox et al., 2019; Guimond-Lischer et al., 2016; Ullah et al., 2020; Fielding et al., 2012)
	Ion implantation	Increased wear resistance Favourable results from *in vivo* animal studies Low temperature technique	Expensive Coatings produced are relatively thin	(Jemat et al., 2015; Hanawa et al., 1997; Qiao et al., 2015; Soares et al., 2018; Zhao et al., 2007)
	Sol-gel	Low cost Low temperature technique Coat complex geometries with ease	Delamination Coatings produced are relatively thin “Soft” coating may pose mechanical stability issues	(Asri et al., 2016; Ferraris and Spriano, 2016; Guo et al., 2015)
	PVD	Increased wear resistance Coat complex geometries with ease	Cost Delamination	(Hong et al., 2010; Ewald et al., 2006; Hauschild et al., 2015; Bergemann et al., 2017; Tanaka et al., 2011; Shunzhi et al., 2018)
	CVD	Increased wear resistance Favourable results from *in vivo* animal studies Coat complex geometries with ease	Cost Delamination	(Giavaresi et al., 2004; Nakai et al., 2015; Martin et al., 2007)
Non-coating	Mechanical methods	Simple Widely used	Further modification often required	(Jager et al., 2017)
	Acid etching	Increased surface energy Removes contamination	Residual ion deposition	(Bhosle et al., 2016; Ferraris and Spriano, 2016)
	Laser	Increased wear resistance High reproducibility Fast Minimal contamination	Cost	(Chan et al., 2017; Coathup et al., 2017; Chien et al., 2014; Mukherjee et al., 2015, 2017; Chen et al., 2011)
	Anodization	Favourable results from *in vivo* animal studies Increased wear resistance Easy to modify parameters	Uniformity issues	(Roy et al., 2011; Kim et al., 2013; Popat et al., 2007; Tsuchiya et al., 2012; Feng et al., 2016)

### Coating Methods

Coating methods are typically employed to introduce an antimicrobial agent and/or a compound that enhances osseointegration. For instance, functionalization of titanium femoral implants with bone sialoprotein can be used to promote bone formation in rat calvarial models when attached with a (3-aminopropyl)triethoxysilane linker (Baranowski et al., 2016).

#### Titanium Nitride (TiN) Coatings

TiN is a hard, biocompatible ceramic coating with impressive corrosion resistance that can be formed on the surface of implants. It is a particularly attractive option from an odontological perspective due its characteristic golden color, which is more subtle and aesthetically acceptable as a dental implant than the gray Ti color, which can strongly contrast the pink nature of gingival tissues (Chouirfa et al., 2019). Several studies have documented an antibacterial effect exhibited by TiN, *Scarano et al.* demonstrated a significant reduction in bacteria occurred in volunteers with TiN coated Ti dental implants in relation to uncoated Ti volunteers (Scarano et al., 2003; Zhao et al., 2009). Conversely, a double-blind randomized control trial found no clinical benefit to TiN-coated CoCrMo in total knee arthroplasty (Van Hove et al., 2015a). It is important to note that the two procedures described here are not directly comparable, highlighting the need for further studies on TiN-coated titanium implants in an effort to clarify conflicting reports.

#### Plasma Spraying

Plasma spraying involves the thermal deposition of a coating onto the titanium surface, most commonly hydroxyapatite (HA), a natural component of bone that helps to enhance osteoconductivity. Furthermore, *in vitro* studies found that the HA coating vastly improves bone growth, and several products are in fact marketed that employ the plasma-sprayed HA coating technology (Shahali et al., 2017; Sun, 2018).

The main drawback associated with plasma spraying method is the temperature required to achieve deposition may disrupt the crystalline apatite structure of the implant, leading to mechanical instability when implanted (Fox et al., 2019). Plasma-sprayed coatings made of HA, incorporating antibacterial agents, have been investigated as anti-infective, biocompatible coatings for orthopedic materials (Guimond-Lischer et al., 2016). Most frequently this approach has utilized silver as the antimicrobial agent, but others investigated include strontium and zinc (Ullah et al., 2020; Fielding et al., 2012).

#### Ion Implantation

In this technique, ions are accelerated in an electrical field toward the titanium substrate and are implanted upon the surface. It has been shown to improve the wear resistance of the biomaterial, subsequently reducing the generation of wear debris *in situ* (Jemat et al., 2015). Ca^2+^-ion-implanted titanium was surgically implanted into rat tibia and highlighted a higher propensity for bone to form on the Ca^2+^-ion-implanted side compared with an untreated titanium sample (Hanawa et al., 1997). Dental *in vivo* studies using dogs found that Ag-embedded titanium implants prepared by plasma immersion ion implantation demonstrated enhanced osteogenesis and increased bone mineral density than the sand-blasted, acid-treated samples (Qiao et al., 2015).

Soares et al. (2018) have investigated low-energy ion implantation to incorporate silver into the surface of titanium plates, with the aim of negating bacterial contamination and biofilm formation. Negligible cytotoxic effect was observed against the MG-63 osteosarcoma cell line; however, the antibacterial properties of the materials are inconclusive. A qualitative agar diffusion methodology was used wherein Ag-implanted samples were placed on *E. coli* spread plates and incubated for 48 h. After this time, no growth was observed directly under the sample, but no extending zone of inhibition was measured (Soares et al., 2018).

Ion implantation has been applied to stainless steel, which, similar to titanium, is a commonly used material for orthopedic devices. N^+^, O^+^, and SiF_3_^+^ ions were implanted into stainless steel 316L discs, with *in vitro* bacterial adherence studied under both static and flow conditions. The study revealed that SiF_3_^+^-implanted steel proved to be more effective than N^+^- and O^+^-implanted steel. This is explained by the lower surface energy and lower surface roughness of SiF_3_^+^-ion-implanted stainless steel surfaces (Zhao et al., 2007). Ion implantation as a technology is well backed by the literature; however, clinical trials are required to evaluate the long-term prospects of this method. In addition, coatings produced by ion implantation are generally thinner than other methods discussed, which may have ramifications for this technique's ability to provide long-term wear resistance and therefore lifespan of the implant.

#### Sol-Gel Deposition

Sol-gel surface modification is a method by which a sample is typically dipped into a colloidal solution (sol), which gradually undergoes polycondensation, leading to the formation of a thin gel layer on the surface. This is then followed by a drying process to remove excess liquid. This widely utilized method is highly robust in that it is able to coat complex shapes such as orthopedic implants with ease (Asri et al., 2016). In addition, this method is low cost and requires low processing temperatures, which help to maintain the bulk mechanical properties of the material (Ferraris and Spriano, 2016).

The sol-gel method has been used to apply a porous, silver nanoparticle-containing coating onto titanium surfaces. This has been shown to reduce the adherence of *S. aureus* by 99.3% after 24 h (Guo et al., 2015). Sol-gel MgO films formed upon titanium substrates exhibit biocompatibility with osteoblast cells *in vitro,* while possessing slight antibacterial effects against *E. coli* (Shunzhi et al., 2018).

#### Physical Vapor Deposition

**Figure 8:**
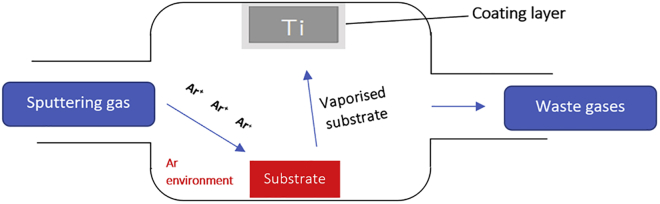
Schematic Showing the Principles Behind Sputtering Physical Vapor Deposition

The term PVD is used to encompass a variety of surface modifications including evaporation, sputtering, and ion plating, the most commonly utilized of which is sputtering. The general premise behind these strategies is that a material is used to form an outer layer on the surface of titanium by initially vaporizing the material and allowing it to condense upon the titanium surface. With respect to orthopedics, the most commonly employed variation is sputtering deposition (seen in Figure 8). Sputtering occurs typically in an argon-rich (Ar) environment, in which a small percentage of the gaseous argon is vaporized into positively charged Ar^+^. Ar^+^ then collides with the chosen substrate, generating a variety of highly reactive substrate molecules that bombard into the titanium and create a coating layer. Magnetron sputtering involves the use of a closed magnetic field to control the rate of ionization, which will influence the coating layer properties.

Studies conducted in this area has yielded promising results. Osteoblast proliferation on hydroxyapatite-coated substrates produced by right angle magnetron sputtering were assessed by Hong et al. The preliminary work highlighted that the samples with sputter produced hydroxyapatite coatings had the highest osteoblast cell density (Hong et al., 2010).

PVD has also been investigated as a technique to produce antimicrobial coatings on titanium-based orthopedic materials. It is widely accepted that silver possesses antimicrobial properties. Titanium wire coated with a silver multilayer coating showed >4 log reduction when tested using a proliferation assay, compared with the untreated titanium against methicillin-sensitive *S. epidermidis* (Fabritius et al., 2020). Titanium/silver hard coatings have demonstrated reduced adherence of both *S. aureus* and *K. pneumoniae*, while displaying no cytotoxic potential *in vitro*. It is thought that the antimicrobial effects of the TiAg coating is due to the release of silver ions (Ewald et al., 2006). PVD-silver-coated titanium joint prostheses have also been assessed in *in vivo* canine models, to address concerns over potential silver toxicity and inhibition of osseointegration. The study revealed stable osseous integration of 4 out of 9 implanted devices, with no local or systemic side effects (Hauschild et al., 2015).

Titanium-copper-nitride (TiCuN) coatings have been deposited on bulk titanium, and this was shown to inhibit biofilm formation on orthopedic implants *in vitro*. The authors of this work suggest that the TiCuN offered osteoblasts a surface adherence advantage and so could out-compete *S. epidermidis* cells in the “race for the surface” (Bergemann et al., 2017).

The major limitation of this method is the initial capital investment required for a sputtering deposition system. Few research groups are currently investigating this technique and therefore the field is still quite niche. Concerns have been raised surrounding the propensity for the coating layer to undergo delamination, in which the surface layer fractures and mechanical failure occurs (Tanaka et al., 2011). Further experimentation into this area is required if it is to garner sufficient commercial interest required for investment.

#### Chemical Vapor Deposition

**Figure 9:**
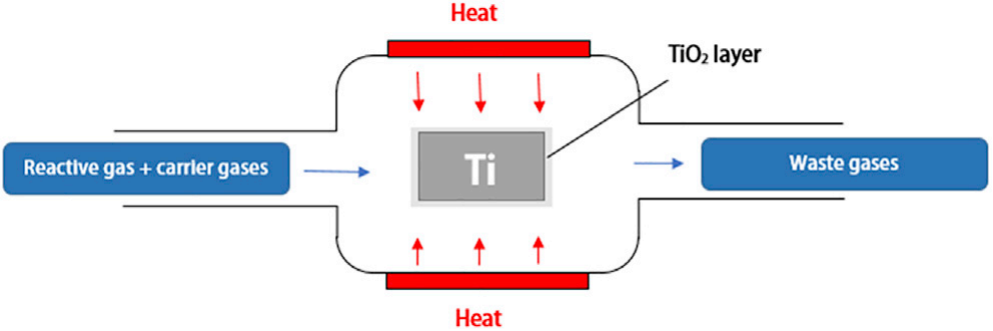
Basic Principles of a CVD Reactor with a Titanium Substrate Showing the Formation of a Protective Layer (in this case TiO_2_)

Chemical vapor deposition, as illustrated in Figure 9, places a reactant gas in a pressurized, high temperature reactor, which allows for the gas to react with the surface of titanium and form a thin protective coating. Although this method requires highly specialized equipment that requires a high initial investment, it is capable of producing a highly uniform coating on complex geometries. Furthermore, the precursor temperature used in CVD can be used to alter the morphology and thickness of the coating formed, making it highly versatile for commercial use (Nakai et al., 2015). Because of the similarity in technique to PVD, delamination also poses an issue for surface modification of materials using CVD, which must be taken into consideration when applying this technology to orthopedic devices.

Experimental success has been demonstrated for titanium implants coated with a TiO_2_ layer via CVD, improving the rate of osseointegration in rabbit in femoral bone at 4 weeks and at 12 weeks (Giavaresi et al., 2004). Coatings of antimicrobial compounds such as poly(dimethylaminomethyl styrene) have been also successfully deposited using CVD, achieving a 4 log reduction in viable *E. coli* (Martin et al., 2007)*.*

### Non-coating Methods

The vast majority of non-coating methods manipulate the roughness of the surface layer of titanium. Surface roughness strongly influences not only osseointegration but also bacterial adhesion. A study by Colon et al. demonstrated that TiO_2_ with a nanostructured surface displayed reduced *S. epidermidis* adhesion (69%) compared with its microstructured TiO_2_ counterpart (Colon et al., 2006). The topography of the bio-implanted substrate will influence the cytoskeletal organization of the cells adjacent to the implant, which alters the downstream biochemical signaling pathways responsible for ossification to occur (Pap et al., 2001).

Bactericidal surfaces may not kill all viable bacteria present at the implant microenvironment, especially if there is no direct contact between the cell and the material surface. However, the initial bacterial attachment, and subsequently bacterial viability, can be influenced by textured surfaces compared with polished surfaces. Prevention of initial attachment through topographical modification can therefore be synergistically utilized alongside bactericidal methods as a means of further reducing the risk of orthopedic infections.

#### Mechanical Methods

Some of the simplest and most commonly employed methods to induce changes in surface roughness include machining, blasting, and grinding. These methods all rely on the mechanical alteration of the titanium surface to form a specific topography, which demonstrates superior osseointegration and/or reduced bacterial adhesion. A problem frequently encountered with these techniques is that other materials used to achieve surface modification are often embedded within the titanium surface. This has a myriad of implications for biomedical applications and as such, mechanical roughening is often used as an initial surface modification prior to a subsequent alteration (Jager et al., 2017).

#### Acid Etching

Acid etching is another process that is used to remove impurities from the titanium surface, leaving behind a clean, homogeneous oxide layer, which possesses an increased surface energy and is able to improve implant integration. Acid etching of cp-Ti and Ti6Al4V, followed by subsequent enrichment with silver ions, produces a silver embedded oxide layer upon the surface. The material in question demonstrated antibacterial and bioactive properties (Ferraris et al., 2014). Although this method is commonly explored in the literature, a major problem with this method is its tendency to deposit residual ions upon the titanium surface, which have the potential to leech into the adjacent tissue (Bhosle et al., 2016).

#### Laser Engineering

Laser surface modification is an emerging technique that has been utilized to facilitate bone apposition and prevent bacterial adhesion. Laser surface modification has a variety of attractive features over conventional mechanical/chemical methods and can be achieved at a much faster rate with high repeatability. These attributes lend laser engineering to be a commercially viable platform for surface modification.

We have previously demonstrated fiber laser surface modification of CP-Ti and Ti6Al4V using a continuous wave (CW) 200W fiber laser system at 1064nm, reducing the initial bacterial adhesion of *S. aureus* and also exerting a bactericidal effect (Chan et al., 2017). Other studies have shown laser-texturing of Ti6Al4V using a Nd:YVO_4_ laser with a wavelength of 532nm to improve bone implant contact to a similar level as hydroxyapatite-coated and grit-blasted implants in ovine models (Coathup et al., 2017), and laser engineering as a technique used to affix biocompatible compounds such as hydroxyapatite to the surface of the orthopedic implant as a means of improving osseointegration (Chien et al., 2014; Mukherjee et al., 2017).

Laser modification is compatible with other techniques as Chen et al. demonstrated by showing laser grooved Ti6Al4V with an RGD coating improved adhesion and bone growth, in comparison to when the individual techniques were employed (Chen et al., 2011). Furthermore, pulsed laser melting of Ti6Al4V can positively influence growth of osteoblast-like MG63 cells (Mukherjee et al., 2015).

As described above, laser surface modification is a viable method as a means of improving the antibacterial performance of titanium-based implants and facilitating osseointegration; however, this is still an emerging area of research.

#### Electrochemical Anodization

Electrochemical anodization is a popular surface modification method in which TiO_2_, which is formed via electrolytic oxidation of the titanium substrate, reacts with the electrolyte media causing TiO_2_ nanotubes to self-assemble on the surface. The ability to alter to properties of the TiO_2_ nanotubes by changing the environmental conditions is of huge benefit to optimizing the surface of titanium (Roy et al., 2011). The TiO_2_ layer formed by anodization is thicker (_∼_200nm) than that which forms naturally (_∼_1.5–10nm) (Kim et al., 2013). Not only that, but the TiO_2_ nanotubes mimic the intrinsic topography of the native bone and are thought to promote biomaterial osseointegration.

Histological analysis of TiO_2_ nanotubes demonstrated increased osteoblast activity *in vivo* using male Lewis rats at 4 weeks (Popat et al., 2007). In another study, TiO_2_ nanotubes were fabricated via anodization, subsequently loaded with gentamicin, before finally being covered in a film composed of gentamicin/chitosan. The antibiotic-loaded nanotubes demonstrated antibacterial effects against *S. aureus,* whereas samples treated with the chitosan film offered reduced *in vitro* cytotoxicity, potentially due to the highly biocompatible nature of chitosan (Feng et al., 2016).

Another innovative approach by Tsuchiya et al. involved a clinical trial in which patients with a post-operative orthopedic infection were treated using titanium implants with an iodine-embedded oxide layer formed via anodization. Upon follow-up, all infections were successfully treated and excellent bone ingrowth was evident on all implants, with no abnormalities in thyroid function noted (Tsuchiya et al., 2012).

In such applications long-term assessment of the mechanical stability of the TiO_2_ layer formed will need to be undertaken to ensure delamination and subsequent release of problematic wear particles is minimized.

## Future Directions and Concluding Remarks

### Bulk Structure

Although this review addresses approaches for surface modification, we have not ignored the bulk structure of the biomaterial and the mechanical requirements that are needed to ensure implant success. Recent innovations intending to mimic the elastic modulus of cortical bone in an effort to prevent bone resorption via the addition of β-stabilizers to titanium have proven to be successful (Chan et al., 2016; Donaghy et al., 2019, 2020). Other research has focused on improving the porosity of the implant structure, again with the intention of mimicking the intrinsic porosity of bone and has shown to improve bone-implant attachment (Pałka and Pokrowiecki, 2018). Another highly interesting area of research is the use of additive manufacturing methods, which have paved the way for biomaterials scientists to accurately manufacture implants with complex geometries tailored uniquely to every patient (Mok et al., 2016).

### Current “State of the Art” and Developments in Surface Modification

Currently the surface modification technique that has shown the most commercial success has been plasma spraying (Sun, 2018; Shahali et al., 2017). Problems encountered with metal-on-metal hip implants have stressed the importance of ensuring a medical device is thoroughly tested before it reaches a widespread clinical setting (Pandit et al., 2008). From the author's perspective, it appears that the discussed methods have all demonstrated promising *in vitro* and, in some cases, *in vivo* results. However, what appears to be limiting the progression of the field is commercial integration and thorough clinical evidence.

Any of the aforementioned surface technologies discussed should ideally be highly reproducible if it is to have the scalability required to reach a commercial setting. In this regard, it is understandable as to why plasma spraying has been commercially viable. Laser surface modification in this respect is another promising method due to its fast, robust, and reproducible nature. Furthermore, with an expanding understanding of the biological processes that govern implant success, there is a growing potential for the use of biologically active coatings. It is envisioned that future marketed titanium orthopedic products could synergistically utilize a biologically active coating and other surface technology in an effort to ensure not only osseointegration, but also sufficient antibacterial properties, thus comprehensively possessing the key attributes for an ideal orthopedic biomaterial: *mechanical strength, biocompatibility, and resistance to infection*.

### Concluding Remarks

The authors have discussed in depth the properties that make titanium such an attractive material for orthopedics. Subsequently, we have provided a brief overview of the biological mechanisms involved in implant failure, which includes both biofilm-mediated prosthetic joint infection and non-infectious complications. Following this, we have highlighted and discussed the most commonly investigated surface modification techniques that have been used to optimize the properties of titanium as an orthopedic biomaterial.
